# Immunological Insights into the Causal Link Between Arthritis, Osteoarthritis, and Frailty: An Integrated Analytical Study

**DOI:** 10.7150/ijms.104476

**Published:** 2025-01-06

**Authors:** Shuyang Wen, Yuxin Cai, Qi Zhang, Baizhi Qiu, Yuting Zeng, Shuqi Zheng, Zhishan Ling, Yupeng Xiao, Pengcheng Lu, Peng Zheng, Na Chen, Guozhi Huang, Qing Zeng, Jihua Zou

**Affiliations:** 1 Department of Rehabilitation Medicine, Zhujiang Hospital, Southern Medical University, Guangzhou, China.; 2 School of Nursing, Southern Medical University, Guangzhou, China.; 3 School of Rehabilitation Medicine, Southern Medical University, Guangzhou, China.; 4 Faculty of Health and Social Sciences, Hong Kong Polytechnic University, Kowloon, Hong Kong SAR, China.

**Keywords:** Rheumatoid arthritis, Osteoarthritis, Frailty, Circulating immune cells, Bioinformatics, Mendelian randomization

## Abstract

**Background:** Previous observational studies have observed associations between rheumatoid arthritis (RA), knee osteoarthritis (KOA), hip osteoarthritis (HOA), and frailty, but the causal relationships remain unestablished.

**Objective:** This study aimed to evaluate the causal relationships between RA, KOA, HOA, KneeHipOA, and frailty using Mendelian randomization (MR) and bioinformatics analysis.

**Methods:** We performed two-sample MR to test for causality between RA, KOA, HOA, KneeHipOA, and frailty. Subsequently, we combined our results in a meta-analysis and conducted multiple sensitivity analyses (MR-Egger, weighted median, constrained maximum likelihood and model averaging (cML-MA), and Bayesian weighted MR (BWMR)). We further explored the role of circulating immune cells and the effects of RA and OA-related gene expression on frailty.

**Results:** Genetically determined RA, KOA, HOA, and KneeHipOA were correlated with a higher risk of frailty. The results of multivariate MR analyses were consistent with those of two-sample MR. Gene Ontology enrichment analysis and Kyoto Encyclopedia of Genes and Genomes analysis indicated that RA and OA-related genes were primarily enriched in various immune responses. Our findings suggested that increases in monocyte cell AC, eosinophil cell AC, and neutrophil cell AC were associated with a higher risk of frailty.

**Conclusion:** This research provides evidence supporting the associations between RA, KOA, HOA, KneeHipOA, and frailty. It also highlights the significant role of circulating immune cells in the development of frailty, indicating the importance of frailty management from an immunological perspective.

## Introduction

Rheumatoid arthritis (RA) is a chronic inflammatory autoimmune disease that leads to the destruction of articular cartilage, bone erosion, and impairment in joints. The global incidence ranges from 0.5% to 1%, decreasing from the northern to the southern hemisphere and from urban to rural areas. It will carry a substantial burden for both the individual and society without timely therapeutic intervention^1^. Osteoarthritis (OA) is a degenerative joint disease that causes disability and health burdens in older adults. It is becoming more prevalent due to obesity and aging worldwide, affecting nearly 250 million people. Although it can affect nearly the whole joint, knee OA (KOA) and hip OA (HOA) are the most common forms in clinical. A multi-center, longitudinal, prospective observational study demonstrated that subjects with KOA pain have an increased risk of developing frailty^2^. The prevalence of KOA and HOA is reported to be 16%-17% and 6%, respectively^3^. As the world's population continues to age, more and more people will be affected by these diseases in the future.

Frailty is a complex, multidimensional disease with degeneration in bodily and cognitive features in older adults. It is considered a biological syndrome in some research due to reduced reserve and resistance to stressors, resulting from cumulative declines across multiple physiological systems, ultimately causing vulnerability to negative outcomes^4^. It is very common in the older population especially among women, affecting 4-17% of the general population^5^. Although a precise and standard definition of frailty remains uncertain, two conceptual models have been widely used to verify the prevalence of frailty: Rockwood's frailty index of accumulated deficits (FI) and Fried's frailty score (FFS). Both techniques are exceptional predictors of the onset of frailty^5^-^7^. As they are the most widely accepted and validated, numerous cross-sectional studies have employed them to assess the prevalence of frailty. A longitudinal study of RA patients found that the prevalence of frailty in the UK Biobank was 20% and 23% when evaluating it using FI and FFS^8^. Another cohort study conducted in the UK Biobank found that the mean ± SD FI was 0.18 ± 0.08 in patients with RA, whereas it was 0.17 ± 0.09 among patients with OA. The prevalence of frailty, determined by FFS, was 18.6% and 10.0% among individuals with RA and OA, respectively^9^.

Numerous epidemiological studies have found that frailty is related to arthritis, with both influenced by some potential mechanisms such as pain, physical inactivity, and chronic low-grade inflammation^10^-^12^. Chronic inflammation and immune responses are crucial in the development of frailty. A longitudinal study demonstrated a correlation between elevated levels of C-reactive protein and white blood cell counts, and the progression of frailty^13^. Concurrently, an increase in neutrophil counts fostered frailty development, while lymphocyte counts counteracted this effect^14^. However, these findings remain remain debated. Additionally, a prospective cohort study found that patients with RA and OA are more likely to develop frailty. Multimorbidity and specific common morbidities may interact with OA and RA to develop frailty^9^. Preceding research has recognized a causal relationship between frailty and OA, but it remains controversial whether a bidirectional causal association exists^15^. Identifying the causality between RA, OA, and frailty is crucial, as it could be beneficial to understanding the disease etiology and finally facilitate the health management of older people.

Unlike traditional observational research affected by confounding factors, reverse causation, and underlying bias, Mendelian randomization (MR) is a powerful tool for identifying causality by using genetic variations as instrumental variables (IVs)^16^. It can effectively avoid the impact of confounders and reverse causes and eventually attain impartial results^17^. While univariable MR (UVMR) assesses the total causal effect of the interested exposure on the outcome, multivariable MR (MVMR) can assess the direct causal effects of interested exposures on outcomes in the presence of potential confounders^18^. Given the above advantages of the MR method, this study aimed to use MR to investigate the causal relationship between RA, OA, and frailty, as well as the influence of circulating immune cells on frailty development. Additionally, bioinformatics was used to analyze the correlation between differentially expressed genes (DEGs) and frailty.

## Materials and Methods

### Study design

**Figure 1:**
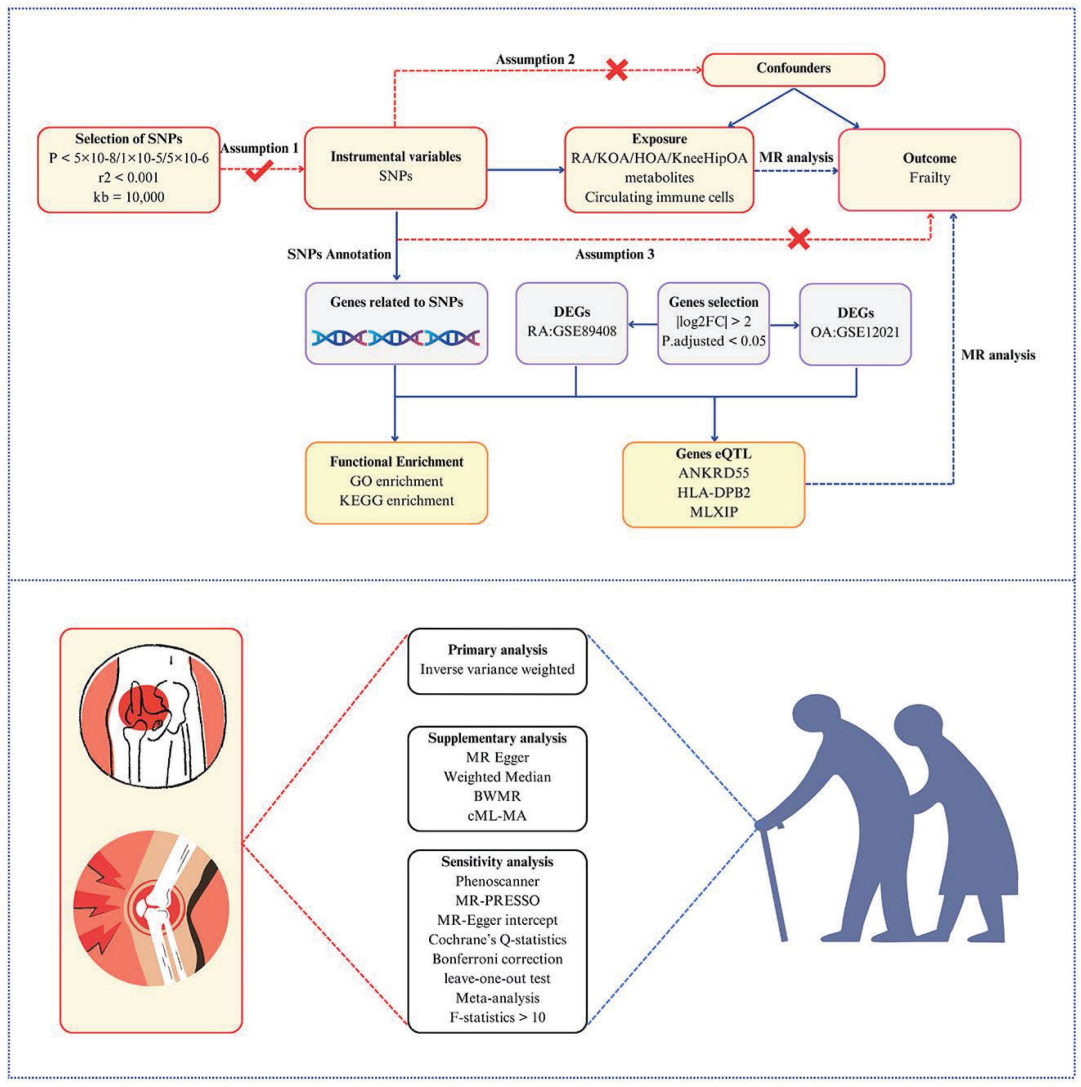
Study overview. Assumptions: 1) the relevance assumption indicated the IVs are tightly related to the exposure; 2) the independence assumption implied that the IVs must be unrelated to any confounders that would potentially influence both the exposure and outcome; 3) the exclusion-restriction assumption required that the IVs purely affect the outcome via the exposure. Abbreviations: RA, rheumatoid arthritis; KOA, knee osteoarthritis; HOA, hip osteoarthritis; KneeHipOA, knee and hip osteoarthritis; DEGs, differentially expressed genes; eQTL, gene expression quantitative trait loci; BWMR, Bayesian weighted Mendelian randomization; cML-MA, constrained maximum likelihood and model averaging.

The study design is briefly summarized in Figure 1. Initially, we employed a two-sample MR to estimate the causal association between RA, OA, and frailty. To obtain more robust and reliable relationships, two-sample MR analyses were carried out using two independent GWAS datasets and the meta-analysis was conducted to assess the combined causality. Additionally, a multivariable MR analysis was conducted to estimate whether the causal association of RA with frailty is robust and reliable after adjusting for some confounders. Genes associated with RA and OA (RAGs and OAGs) were identified through single nucleotide polymorphisms (SNPs) enrichment, and biological processes were discerned via enrichment analysis. Subsequently, the gene expression data of RA and OA patients from the GEO database were utilized to screen differential genes. Gene expression quantitative trait loci (eQTL) were employed to investigate the correlation between gene expression and frailty progression. Lastly, the correlation between circulating immune cells and frailty was examined.

### Data sources

Summary statistical data for frailty (FI and FFS) in the European population were extracted from the latest GWAS studies. The GWAS data for KneeHipOA, KOA, and HOA were downloaded from the Genetics of Osteoarthritis (GO) Consortium (https://www.genetics-osteoarthritis.com) and the GWAS data for RA were from the FinnGen Consortium. The summary statistics of RA, KneeHipOA, KOA, and HOA for replication analysis were obtained from the IEU OpenGWAS Project (https://gwas.mrcieu.ac.uk/).

Circulating immune cells absolute count (AC) data were derived from the Blood Cell Consortium (BCX), which included basophils, leukocytes, monocytes, lymphocytes, eosinophils, and neutrophils^19^. The lymphocyte subtypes, comprised CD4 T cells, CD8 T cells, CD4CD8^+++dim^ T cells, B cells, HLA-DR natural killer (NK) cells, natural killer T cells, and regulatory T cells (both secretory and resting forms), were derived from Valeria Orrò *et al.*^20^.

The GSE89408 dataset for RA and the GSE12021 dataset for OA were downloaded from GEO database (https://www.ncbi.nlm.nih.gov/geo/), which were tested in synovial membrane samples of RA patients, OA patients, and normal controls.

All summary data utilized in this research were publicly available, and detailed information can be found in supplementary table 1.

### IV selection

To select the IVs that adhered to three assumptions, we first screened SNPs tightly associated with the exposure at genome-wide significance (p<5×10^-8^). Secondly, the chosen SNPs were clumped for linkage disequilibrium (LD) in a strict cutoff (kb = 10,000, R2 < 0.001). In addition, to prevent the impact of weak IVs, we also employed the F-statistics to assess the strength of IVs, which is calculated as 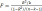 , where *R*^2^ represents the proportion of variation in exposure explained by the IVs, n is the sample size in the original research and k represents the number of IVs. It was regarded as weak IVs if the F-statistics were less than 10 in the MR analysis^21^. We eliminated those SNPs possessing intermediate allele frequencies by harmonizing exposure and outcome data. Finally, all selected SNPs were examined in Phenoscanner to ensure that each SNP was not associated with potential confounders (education; BMI; physical activity; smoking status; drinking; insomnia) (p<5×10^-8^ and R^2^<0.8)^22^.

### Functional annotation

SNP Annotation Tool (https://www.snp-nexus.org/v4/#text_area/c047aa1f) was conducted to functionally annotate to identify genes that are related to SNPs from RA and KneeHipOA by positional mapping.

### Pathway and functional enrichment analyses

Enrichment analyses including Gene Ontology (GO) enrichment analysis and Kyoto Encyclopedia of Genes and Genomes (KEGG) analysis were performed in R software.

### Differential expression analysis

Differential expression analysis for the GSE89408 dataset and the GSE12021 dataset was carried out by GEO2R (https://www.ncbi.nlm.nih.gov/geo/geo2r/) to obtain differentially expressed genes (DEGs). Screening conditions to identify DEGs were |log2FC| > 2 and P.adjusted < 0.05.

### Statistical analysis

We separately obtained the estimated results from the Finngen and GO consortium and performed a meta-analysis to pool the causal relationships in different GWAS datasets. The random-effects inverse variance weighted (IVW) approach was selected as the main analysis to estimate the potential causal effects. As it depends on the hypothesis that three core assumptions in MR analysis are satisfied, the causal effects estimated by IVW might be biased if potential horizontal pleiotropy exists^21^. To validate our findings, we also employed two alternative approaches: Weighted Median and MR-Egger. The weighted median approach could provide consistent results if half of the IVs are valid. The MR-Egger approach could overlook the impacts of the invalidity of IVs and provide a comparatively robust estimate. Nevertheless, both methods could only serve as complementary methods as they may compromise power such as wide confidence intervals (CI)^23^. To verify that our findings were not impacted by potential horizontal pleiotropy, we used the MR-PRESSO global test and the MR-Egger intercept to evaluate the robustness and reliability of the results^24^. The MR-PRESSO outlier test was performed to detect the outlying SNPs. The results would be re-estimated following the exclusion of outlying SNPs. Additionally, to avoid the uncertainty of weak effects owing to horizontal pleiotropy and strengthen the credibility of the results, the methods of constrained maximum likelihood and model averaging (cML-MA) and Bayesian weighted Mendelian randomization (BWMR) were employed to validate the causal relationships^25^, ^26^. The Cochrane's Q-statistics method was utilized to test the heterogeneity between SNPs. The IVW p-value was considered statistically significant when it survived after Bonferroni correction (p<0.05/4=0.0125), with consistent directions in the Weighted Median and MR-Egger. The IVW p-value ranging from 0.0125 to 0.05 was deemed a potential effect. Finally, a leave-one-out test was conducted to assess whether our results were influenced by a single SNP.

All statistical analyses in this research were completely conducted utilizing the TwoSampleMR and MR-PRESSO packages in R software (version 4.3.1) and online tools.

## Results

### Univariable analysis of FI

#### Discovery results of FI

**Figure 2:**
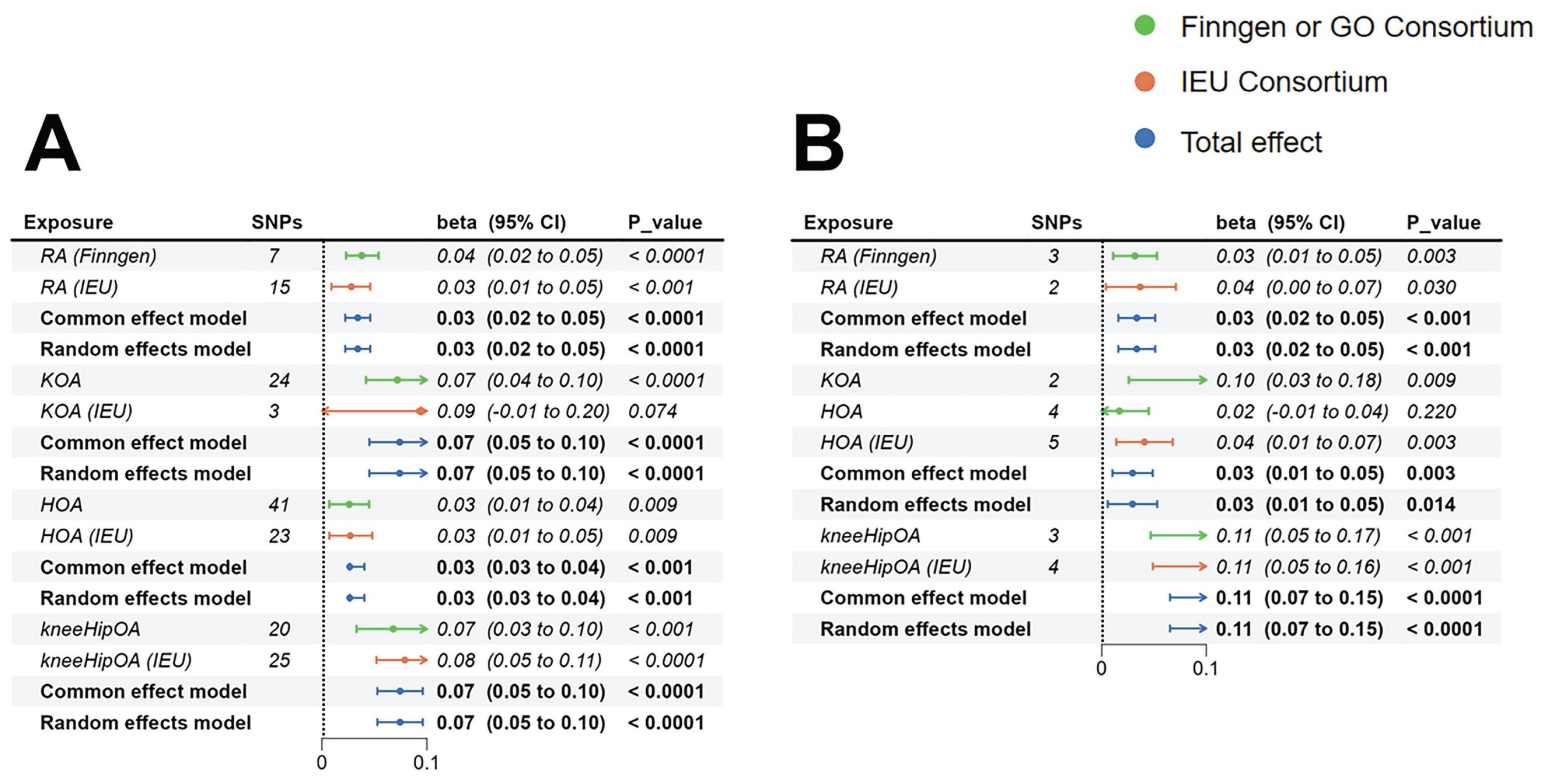
The MR estimates of RA, KOA, HOA, and KneeHipOA on frailty. A: MR estimates of FI; B: MR estimates of FFS. Since there was only one result between KOA and FFS, the method of meta-analysis was not available. Abbreviations: FI, frailty index; FFS, Fried's frailty score; RA, rheumatoid arthritis; KOA, knee osteoarthritis; HOA, hip osteoarthritis; KneeHipOA, knee and hip osteoarthritis.

MR based on the IVW method indicated robust causal links between genetically determined RA (β=0.038, 95%CI:0.023-0.054, p<0.001), KOA (β=0.072, 95%CI:0.042-0.102, p<0.001), HOA (β=0.026, 95%CI:0.007-0.045, p=0.009), KneeHipOA (β=0.068, 95%CI:0.033-0.102, p<0.001), and higher FI (Figure 2).

#### Replication results of FI

To verify the consistency of results across various datasets, we re-analyzed using the IEU database. Our findings indicated that genetically determined RA (IEU) (β=0.028, 95%CI:0.009-0.046, p=0.003), HOA (IEU) (β=0.027, 95%CI:0.007-0.048, p=0.009), KneeHipOA (IEU) (β=0.079, 95%CI:0.052-0.107, p<0.001), and FI exhibited consistent results while the association between KOA (IEU) (β=0.093, 95%CI:-0.009-0.196, p=0.074) and FI can not be confirmed (Figure 2).

#### Combined results of FI from the meta-analysis

Further meta-analysis of the causal association between RA and FI continued to support the notion that RA would increase the risk of FI (β=0.034, 95%CI:0.022-0.046, p<0.001). The results of the meta-analysis between KOA (β=0.074, 95%CI:0.045-0.103, p<0.001), HOA (β=0.026, 95%CI:0.026-0.040, p<0.001), KneeHipOA (β=0.074, 95%CI:0.053-0.096, p<0.0001), and FI also demonstrated that they were risk factors for the development of FI (Figure 2).

#### Sensitivity analyses

**Figure 3:**
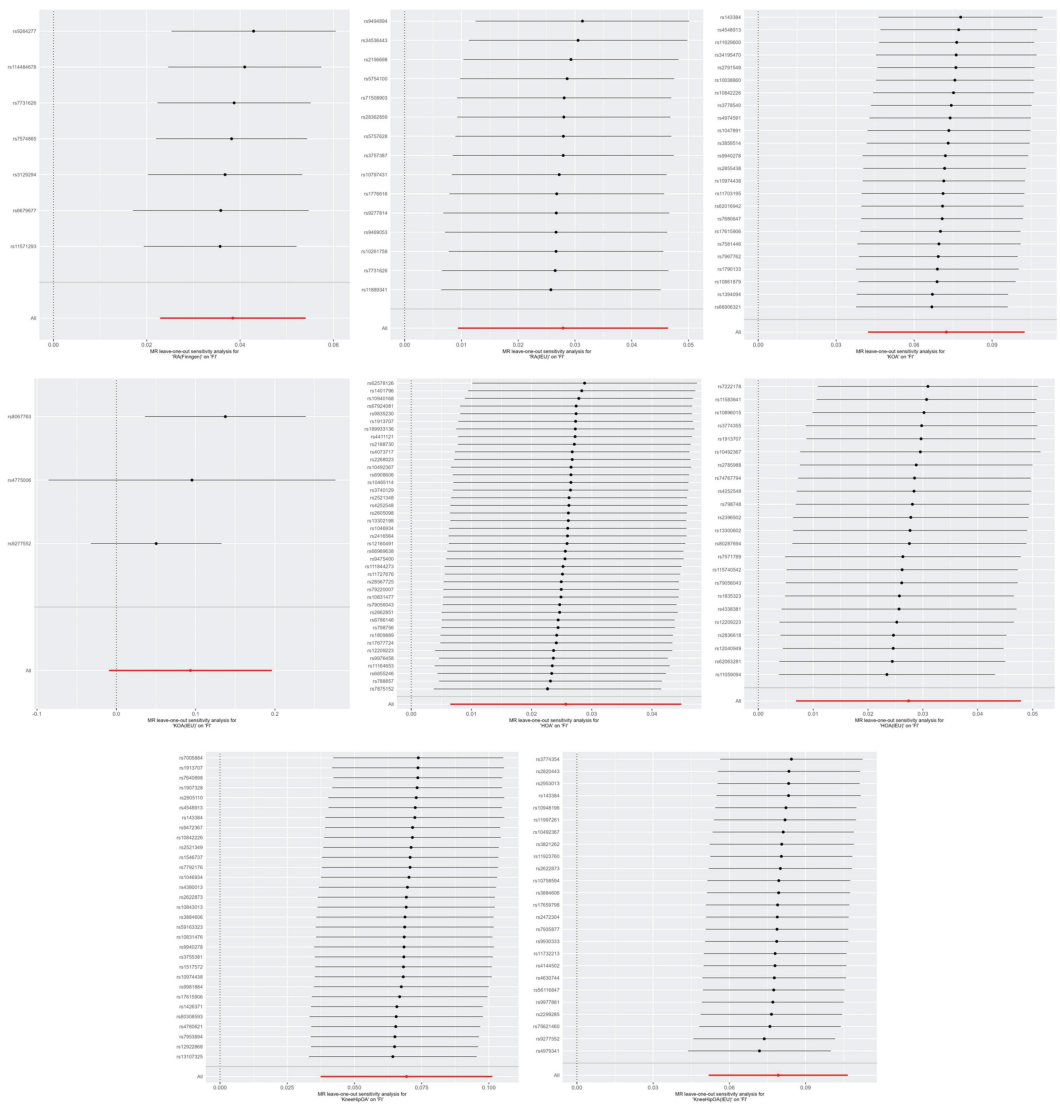
The leave-one-out plots of RA, KOA, HOA, and KneeHipOA on FI. Abbreviations: FI, frailty index; RA, rheumatoid arthritis; KOA, knee osteoarthritis; HOA, hip osteoarthritis; KneeHipOA, knee and hip osteoarthritis.

Similar results were observed using alternative MR methods: the Weighted Median and MR-Egger. The methods of cML-MA and BWMR also yielded the same results, indicating that our analyses were unlikely to be influenced by invalid IVs with only weak pleiotropic effects (Supplementary Table 2). The leave-one-out test showed that none of the SNPs independently affected the estimated causal relationships except the relationship between KOA (IEU) and FI (Figure 3). So we further examined the traits associated with the corresponding SNPs (rs4775006/rs9277552) (Supplementary Table 6). The causal relationships between the above diseases and FI were unlikely to be biased by horizontal pleiotropy effects and there is no heterogeneity (p-values for both heterogeneity and pleiotropy > 0.05) (Supplementary Table 4). For the analysis of KneeHipOA and FI, we eliminated 11 SNPs (F-statistics <10) to eliminate the impact of weak IVs on the final results (Supplementary Table 5). The IVs employed in other analyses all demonstrated F-statistics over 10, indicating the absence of a weak IV problem (Supplementary Table 4).

### Univariable analysis of FFS

#### Discovery results of FFS

MR analysis based on the IVW approach demonstrated that genetically predicted RA (β=0.032, 95%CI: 0.011-0.053, p=0.003), KOA (β=0.103, 95%CI: 0.026-0.18, p=0.009), and KneeHipOA (β=0.107, 95%CI: 0.047-0.166, p<0.001) were significantly associated with higher FFS. However, there was no evidence to suggest a causal relationship between HOA and FFS (β=0.017, 95%CI: -0.01-0.045, p=0.22) (Figure 2).

#### Replication results of FFS

To validate the consistency of our findings across different datasets, we conducted an analysis using the IEU database. We found there were causality between HOA (IEU) (β=0.041, 95%CI: 0.014-0.068, p=0.003), KneeHipOA (IEU) (β=0.107, 95%CI: 0.049-0.164, p<0.001), and higher FFS. What's more, a suggestive causal relationship was identified between RA (IEU) and higher FFS (β=0.037, 95%CI:0.004-0.071, p=0.03) (Figure 2). However, the analysis between KOA (IEU) and FFS could not be conducted due to insufficient independent SNPs.

#### Combined results of FFS from the meta-analysis

The results integrated by meta-analysis from 2 different datasets further confirmed the established causal relationships. Genetically predicted RA (β=0.034, 95%CI:0.016-0.051, p<0.001), HOA (β=0.03, 95%CI:0.01-0.05, p=0.003), and KneeHipOA (β=0.107, 95%CI:0.065-0.148, p<0.001) were significantly associated with higher FFS (Figure 2). The findings between KOA and FFS were unable to be integrated due to insufficient results, so the final causality was based on only one result.

#### Sensitivity analyses

**Figure 4:**
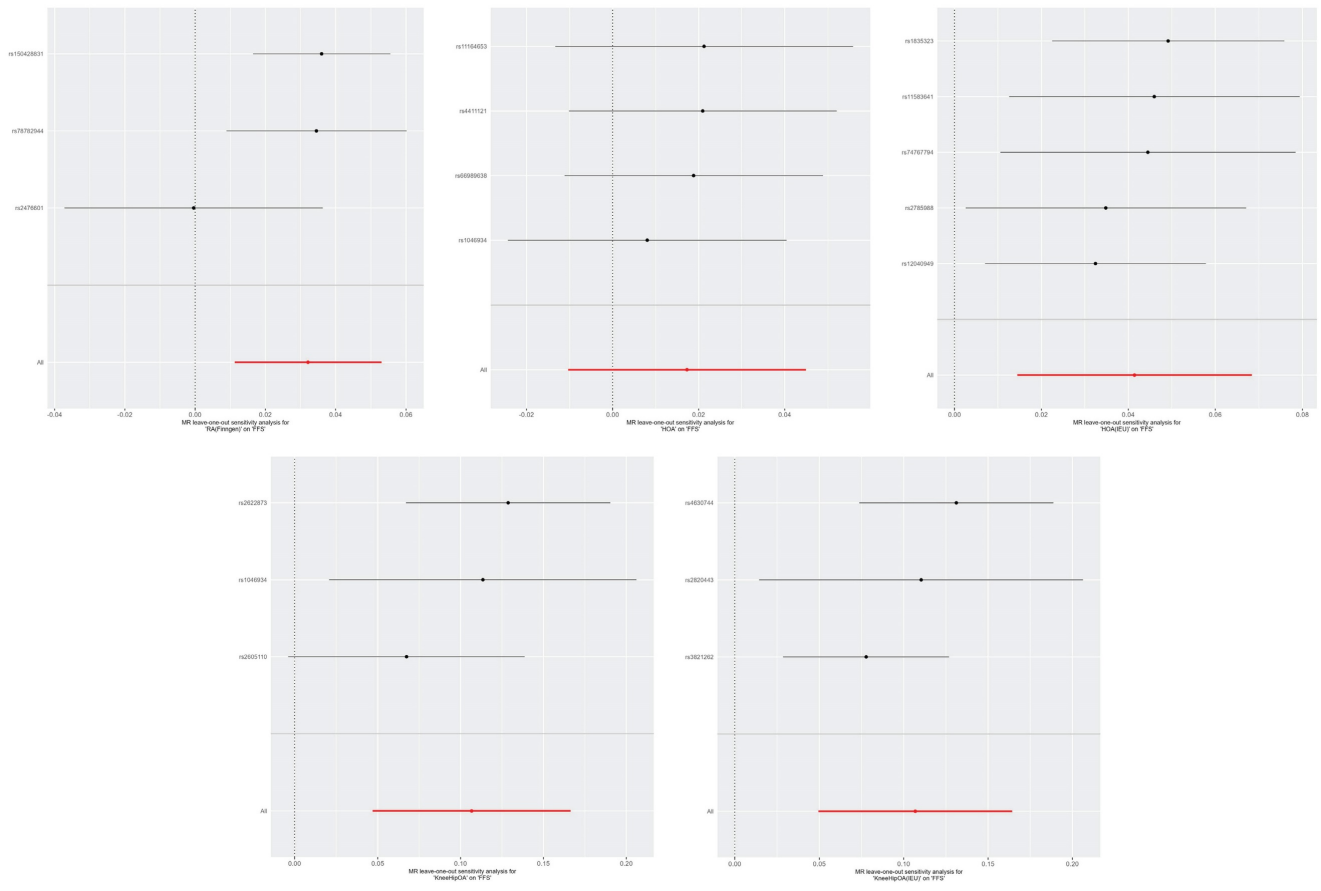
The leave-one-out plots of RA, KOA, HOA, and KneeHipOA on FFS. The leave-one-out plots were not available between KOA, KOA (IEU), RA (IEU), and FFS due to insufficient number of SNPs.

The Weighted Median, MR-Egger, cML-MA, and BWMR methods all yielded similar results (Supplementary Table 4). The leave-one-out method indicated that the relationships between RA, KneeHipOA, and FFS seem to be not robust enough as they were respectively driven by a single SNP (rs2476601/rs2605110) (Figure 4). So, we further screened the traits associated with corresponding SNPs (Supplementary Table 6). The evidence of horizontal pleiotropy effects and heterogeneity for IVs was not found by using the MR-PRESSO global test, MR-Egger intercept, and Cochrane's Q test (Supplementary Table 4). In our analyses, all the F-statistics of IVs were over 10 (Supplementary Table 4).

### Reverse MR analysis

**Figure 5:**
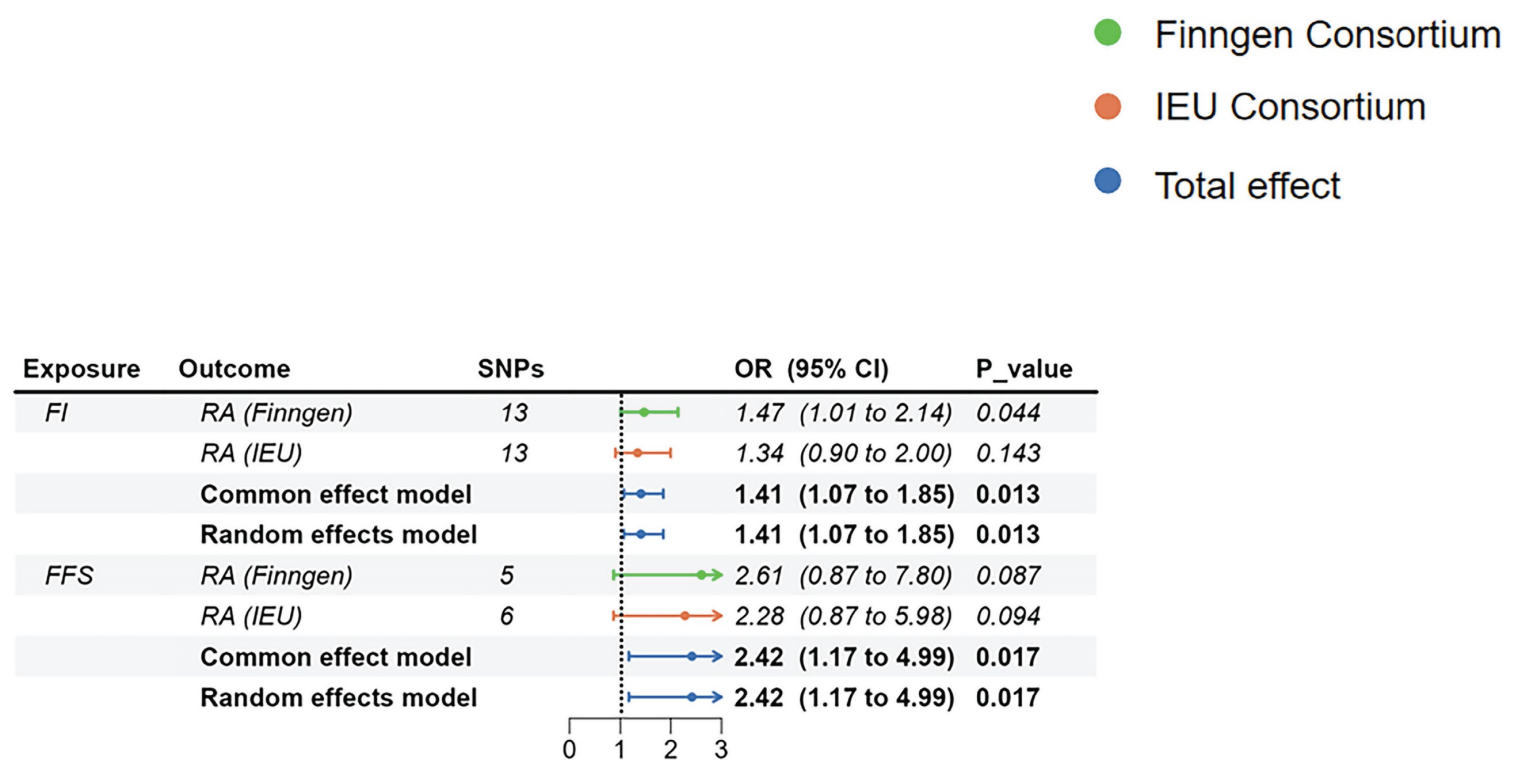
The MR estimates of frailty on RA. Abbreviations: FI, frailty index; FFS, Fried's frailty score; RA, rheumatoid arthritis.

In the reverse MR, we only found a suggestive association between genetically determined higher FI (OR=1.472, 95%CI:1.011-2.144, p=0.044) and the risk of RA (Finngen). There was no enough evidence indicating that genetically determined higher FI and genetically determined higher FFS would increase or decrease the risk of RA (Figure 5). Notably, the F-statistics of IVs between FI, FFS, and RA (Finngen) were all less than 10, suggesting that our results may be affected by weak IVs (Supplementary Table 4). Heterogeneity and pleiotropy were not identified in the above analyses (Supplementary Table 4).

Although there were no significant relationships in different GWAS datasets, the summarized results of the meta-analysis showed that higher FI (OR=1.41, 95%CI: 1.074-1.852, p=0.013) and higher FFS (OR=2.417, 95%CI:1.172-4.985, p=0.017) would increase the risk of RA (Figure 4). Due to the inconsistent results in the reverse MR analyses and the impact of weak IVs, we suggested that the results of the reverse MR should be regarded as exploratory analyses.

### MVMR analysis

MVMR analyses were designed to assess the direct effect of RA on frailty and to adjust for multiple risk factors associated with frailty (including hypertension, T2DM, COPD, OA, BMI, depression, chronic pain, OP, CAD). The results obtained through MVMR were in line with those obtained through UVMR. Genetic liability to RA demonstrated consistent relationships with higher FI and higher FFS (Table 1).

### SNP Annotation and functional enrichment

**Figure 6:**
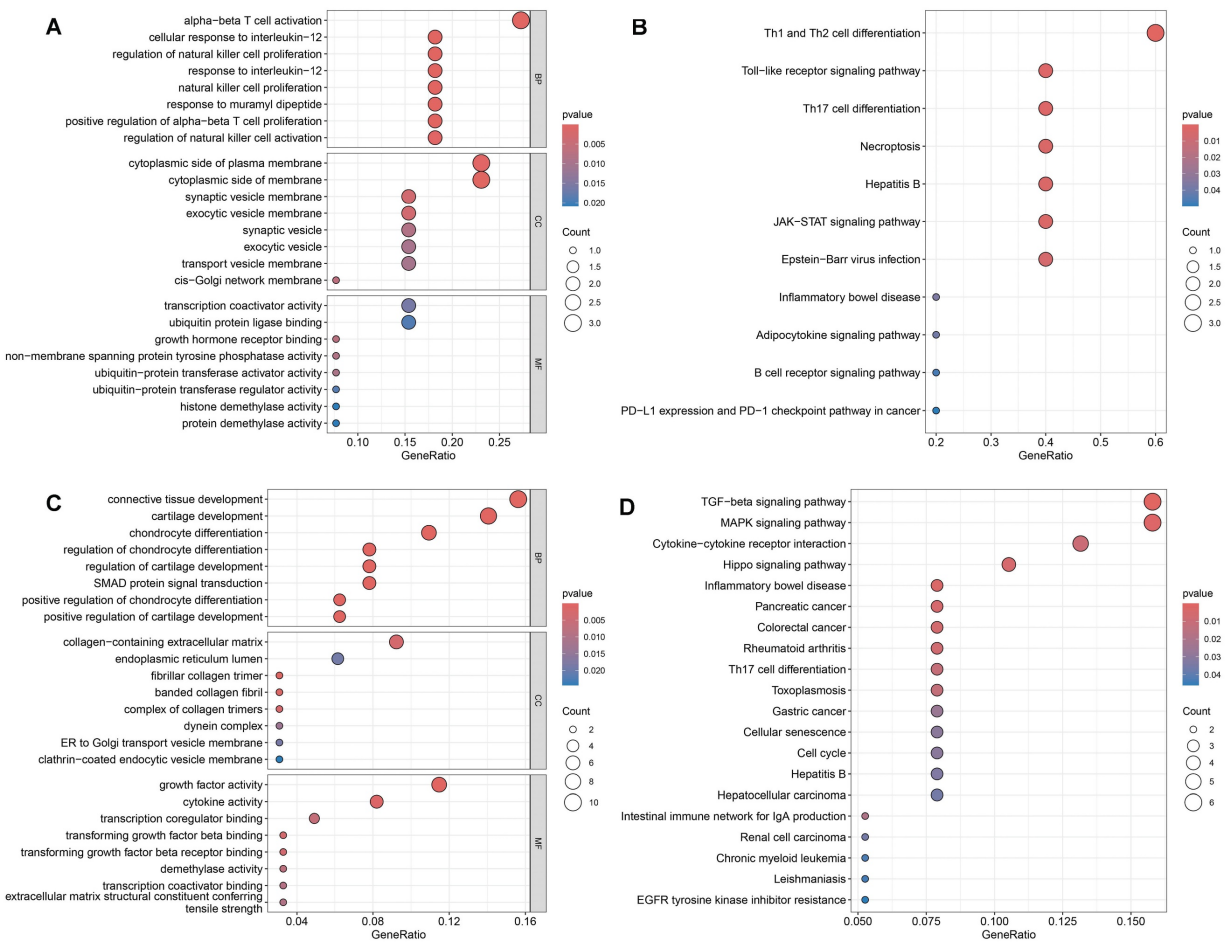
GO and KEGG enrichment analysis. A: The GO results for RAGs. B: The KEGG results for RAGs. C: The GO results for OAGs. D: The KEGG results for OAGs.

Using the SNP Annotation Tool, we identified 21 RAGs and 89 OAGs (Supplementary Table 7). The results for RAGs indicated a primary focus on immune response processes, including alpha-beta T cell activation and cellular response to interleukin-12, among others (Figure 6A). KEGG enrichment pathway analysis suggested significant enrichment in various immune responses, such as Th1 and Th2 cell differentiation and Toll-like receptor signaling pathway (Figure 6B). For OAGs, GO enrichment indicated that these genes were primarily enriched in connective tissue and cartilage development(Figure 6C). KEGG analysis revealed significant enrichment in the TGF-beta signaling pathway, MAPK signaling pathway, and Cytokine-cytokine receptor interaction (Figure 6D).

### Identification of DEGs and eQTL analysis

**Figure 7:**
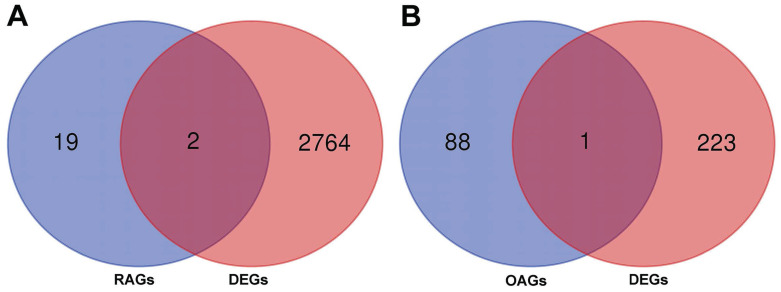
The merged results bewteen RAGs, OAGs and DEGs. A: The merged results bewteen RAGs and DEGs. B: The merged results bewteen OAGs and DEGs.

A total of 2766 DEGs were identified in GSE89408 dataset and 224 DEGs were identified in GSE12021. By merging DEGs, RAGs and OAGs, ANKRD55 and HLA-DPB2 were identified for RA and MLXIP was identified for OA (Figure 7). We therefore utilized the eQTL data to further investigate the relationship of gene expression on the development of frailty. Our results indicated that HLA-DPB2 expression (β=-0.02, 95%CI:-0.03,-0.01, p<0.001) and MLXIP expression (β=-0.02, 95%CI:-0.04,0, p=0.03) were associated with a lower risk of frailty (Table 2).

### Causal estimates of circulating immune cell counts and lymphocyte subtypes

**Figure 8:**
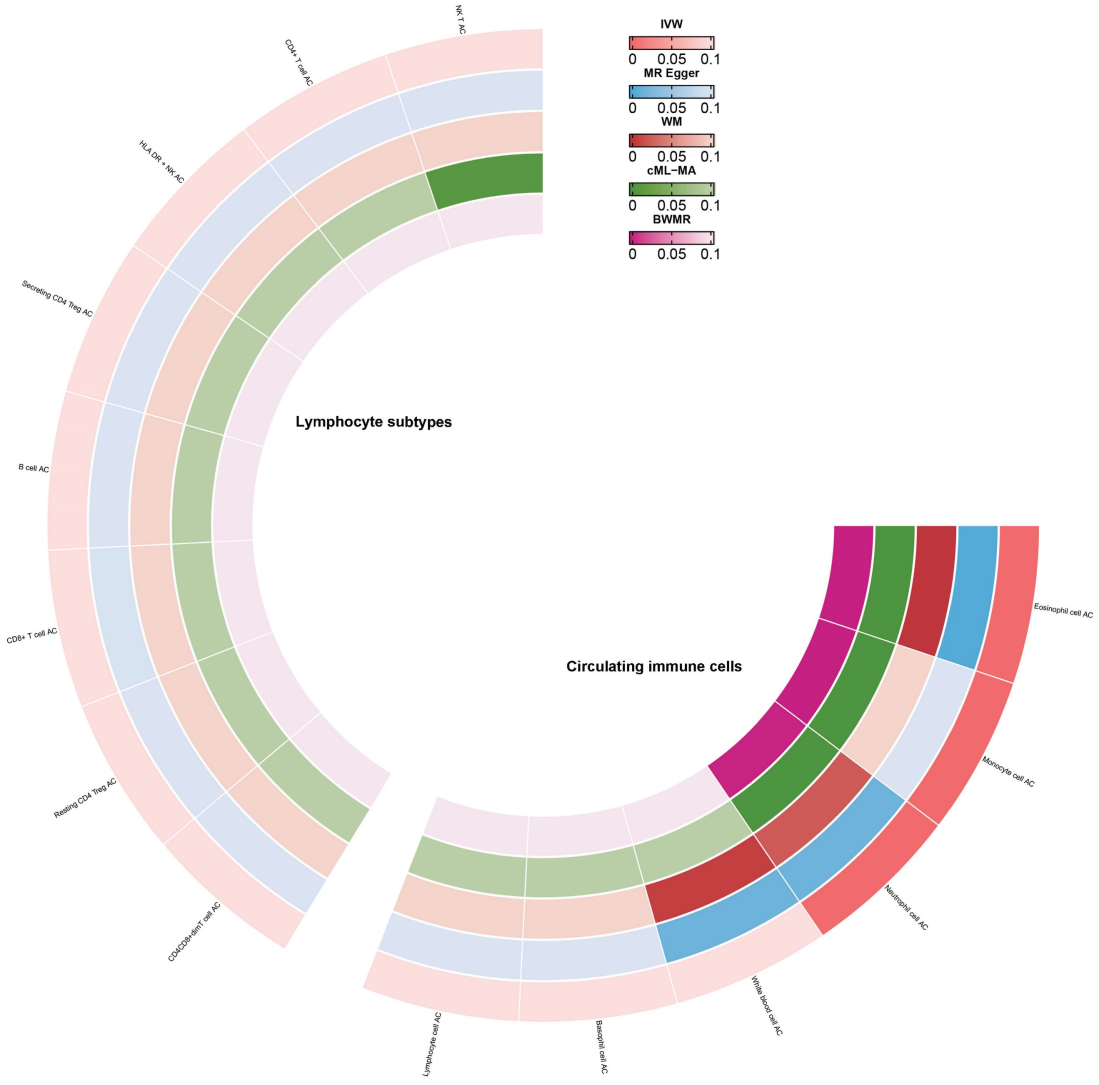
Causal estimates of circulating immune cells on frailty.

Enrichment analysis revealed that RAGs and OAGs were primarily enriched in immune-related processes. Consequently, we delved deeper into the causal relationship between circulating immune cells AC and frailty. Our findings suggested that an increase in monocyte cell AC (β=0.08, 95%CI:0.05,0.1, p<0.001), eosinophil cell AC (β=0.06, 95%CI:0.05,0.08, p<0.001), and neutrophil cell AC (β=0.03, 95%CI:0.02,0.05, p<0.001) was associated with a higher risk of frailty (Figure 8). We observed no significant evidence of a relationship between lymphocyte subtype cell AC and frailty (Figure 8) and there were no significant heterogeneity in our analyses (Supplementary Table 8). But we discovered that horizontal pleiotropy might influence the relationship between monocyte cell AC and frailty (Egger_intercept p<0.001). Lastly, after excluding individual SNPs in the leave-one-out test, the estimates remained stable.

## Discussion

In this research, we used the MR approach to investigate the causal effects of RA and OA on frailty from a genetics viewpoint and conducted confirmatory analyses to support our results. To obtain robust and reliable results, we performed a meta-analysis to combine results from the discovery cohort and replication, confirming the associations between RA, KOA, HOA, KneeHipOA, and frailty. Concurrently, we analyzed the impact of gene expression on frailty and investigated the role of circulating immune cells in its development. Our findings indicated that RA and OA may contribute to frailty development, potentially through immune response. Monocytes, eosinophils, and neutrophils were involved in the occurrence of frailty.

### The correlation between RA, OA, and frailty

Using the latest and large-scale GWAS data, we found positive causal relationships between RA, OA, and frailty. After adjusting for multiple other risk factors for frailty, the association of RA and frailty still remained. To our knowledge, this is the first study utilizing the MR approach for investigating the causal relationship between RA, OA, and frailty (including FI and FFS).

Many current epidemiological research have investigated the correlation between RA, OA, and frailty. For example, various studies have investigated the association between frailty and OA and indicated that assessing frailty status is crucial when contemplating OA treatment, as it may be critical for targeted therapeutic interventions. Different methods measure frailty and consider OA at different sites, all observing a high incidence of frailty in individuals with OA, with unadjusted rates ranging from 24% to 60%^27^-^30^. Notably, a European multi-center study showed that patients with OA at any joint had a 1.5 to nearly 3 times higher risk, and an 8 times higher risk for multiple joints^31^. In a cohort of 100 RA sufferers under the age of 65, 15% were found to exhibit frailty, with an additional 30% considered prefrail^32^. Based on the previous report, we observed causal relationships of RA and OA on frailty using MR analysis. Nevertheless, additional randomized clinical trials are necessary to verify this hypothesis.

### The possible mechanism between OA and frailty

There are several possible explanations for the causal relationship between OA and frailty. On the one hand, patients with OA may develop weakness due to a variety of reasons. Patients with lower limb OA, particularly in the knee and hip, may decrease their physical activity, leading to a reduction in muscle mass and an increased risk of falls^33^, while OA-related pain also causes weakness^29^. The pathways that connect OA with frailty remain unclear and have seldom been studied. Inflammation is considered a possible mechanism involving the development of both OA and frailty. Low-grade inflammation commonly occurs in individuals with OA and some pro-inflammatory markers, such as IL-6 and CRP, have been detected in their blood^34^, ^35^. This chronic and low-grade inflammation can cause sarcopenia and reduce physical activity^36^. On the other hand, frailty could potentially increase the risk of developing OA. Sarcopenia is frequently observed in frail patients, which could result in joint instability and consequently increase the probability of biomechanical damage. Furthermore, frail individuals are at increased risk of falling, which can lead to fractures and disability, ultimately leading to post-traumatic OA^4^. In addition, frail subjects exhibited significant elevations of proinflammatory cytokines, such as IL-6, CRP, and TNF-α^37^-^39^, and it is hypothesized that these mediators may accumulate in the joints and subsequently trigger local low-grade inflammation, catabolic changes, and cartilage destruction in joint structures. Changes in joint cell composition are common in older individuals. Such changes, combined with proinflammatory states that impair joint repair, may lead to the development of OA. As a result, frailty is regarded as another possible risk factor for OA^36^.

### The possible mechanism between RA and frailty

Our results concerning the causal effects between RA and frailty corroborate previous epidemiological research. In addition to affecting multiple joints and causing symmetric arthritis, RA has extra-articular manifestations, including systemic symptoms and involvement of major organs. Patients with RA may exhibit characteristics of weakness, including sarcopenia, fatigue, and reduced physical activity, particularly if the disease is poorly managed^40^, ^41^. In the clinical treatment strategy of RA, the process leading to weakness besides the disease itself may be related to glucocorticoids or anti-rheumatic drugs (DMARDs). Chronic steroid therapy may negatively impact bone and muscle, eventually leading to frailty^42^, ^43^. Glucocorticoids may be beneficial for better disease control during maintenance therapy. Synthetic and biological DMARDs are crucial for steroid retention and should be initiated promptly after an RA diagnosis^44^. Nevertheless, immunosuppression augments the susceptibility to infection, which could result in hospitalization and further frailty^45^. However, the causal model of RA, OA, and frailty is currently very complex. For various reasons, it is necessary to study the specific mechanisms between RA, OA, and frailty.

### Circulating immune cell counts and FI

Our findings emphasize that an increase in monocytes, neutrophils, and eosinophils can contribute to frailty development. As reported earlier, frail patients had a higher proportion of monocytes in their peripheral blood than the normal population, and there was a high expression of long non-coding RNAs such as NEAT1 and MALAT1^46^. Monocytes might collaborate with chronic low-grade inflammation to induce frailty. It has been observed that frailty related to aging was closely linked with chronic low-grade inflammation and monocyte activation^47^. Concurrently, studies have verified that under prolonged inflammatory exposure, monocytes can express large amounts of CRP and TNF^48^. Results from the Women's Health and Aging Studies I also confirmed the correlation of neutrophil cell and monocyte cell counts with frailty in women with disabilities in the community. This correlation could be due to the significant role both neutrophils and monocytes play in inflammation and immune response. Both could produce numerous cytokines, such as IL-6, to regulate inflammation, which can lead to frailty^49^. Additionally, observational studies suggested that eosinophils were not linked with frailty. However, our genetic results supported the idea that increased eosinophil cell counts can cause frailty. While the specific mechanism is yet to be revealed, it might also be involved in inflammation and immune responses. Similar to neutrophils and monocytes, eosinophils play a crucial role in inflammatory activation. Their over-activation would lead to increased inflammatory responses and tissue damage^50^, ^51^. Regardless, our results underscore the role of circulating immune cells in frailty development.

### Strengths and limitations

This study exhibits several advantages: At first, we examined the causality of RA, KOA, HOA, KneeHipOA, and frailty using the UVMR approach and explored the reverse relationship of RA and frailty, MR approach is less susceptible to confounders, reverse causality, and non-differential exposure compared with observational studies^52^. Second, independent GWAS datasets related to RA and OA were used for discovery analysis and replication analysis, and meta-analysis was performed to ensure the reliability of the results. Additionally, this is the first MR analysis to use GWAS data scored by different assessment tools to represent frailty status. While FI and FFS have significant overlap in identifying frailty, their assessments differ in content, thus confirming the reliability of the results from multiple perspectives^53^, ^54^. Finally, we identified genes associated with frailty through bioinformatics and highlighted the role of immune-inflammation in frailty.

Notwithstanding these advantages, it is important to consider several limitations when interpreting our findings. 1) As the GWAS summary data used in this analysis was obtained solely from the European population, our findings are difficult to generalize to other populations. 2) Previous studies found that women with arthritis were more likely to develop frailty, frailty is also more likely to occur in women^22^, ^36^. However, the stratified analysis cannot be performed due to the lack of suitable summary-level data. 3) The magnitude of the provided estimates cannot be directly interpreted in the same way as classic observational studies. We must acknowledge that the estimates in MR analysis reflect the average lifelong effect of the genetically predicted exposure. Therefore, it is more reasonable to determine the direction of the relationship rather than quantifying the magnitude of the estimates^55^. 4) the IVs employed between frailty and RA (Finngen) are weak instruments, which may reduce the statistical power of the results. Caution is needed when applying the results, and we believe it is more appropriate to regard them as exploratory studies.

## Conclusion

In conclusion, this study supports associations between RA, KOA, HOA, KneeHipOA, and frailty. Our results highlighted the potential of circulating immune cell count serving as novel biomarkers for assessing the development of frailty status. Based on our findings, considering appropriate RA and OA management is critical to reduce the risk of frailty. In frail patients, it is necessary to pay close attention to functional decline resulting from inflammation and immune responses. Incorporating the frailty concept into RA and OA care may augment healthcare professionals' comprehension of patients' holistic health, cutting-edge risk assessment, and more personalized treatment approaches.

## Supplementary Material

Supplementary tables.

## Figures and Tables

**Table 1:** MVMR results between RA and frailty

Outcome	beta (95%CI)	P_value
FI		
Adjusted for hypertension	0.03 (0.013,0.047)	3.98E-04
Adjusted for T2DM	0.038 (0.02,0.057)	3.19E-05
Adjusted for COPD	0.042 (0.028,0.056)	7.95E-09
Adjusted for OA	0.037 (0.022,0.051)	9.48E-07
Adjusted for depression	0.042 (0.014,0.07)	3.60E-03
Adjusted for chronic pain	0.042 (0.032,0.051)	1.51E-17
Adjusted for BMI	0.026 (0.009,0.044)	2.81E-03
Adjusted for OP	0.04 (0.023,0.057)	4.06E-06
Adjusted for CAD	0.042 (0.021,0.063)	6.87E-05
FFS		
Adjusted for hypertension	0.03 (0.015,0.046)	1.36E-04
Adjusted for T2DM	0.028 (0.011,0.045)	1.46E-03
Adjusted for COPD	0.047 (0.045,0.049)	0.00E+00
Adjusted for OA	0.033 (0.001,0.066)	4.42E-02
Adjusted for depression	0.03 (0.007,0.053)	1.20E-02
Adjusted for chronic pain	0.032 (0.011,0.053)	2.62E-03
Adjusted for BMI	0.03 (0.014,0.045)	1.47E-04
Adjusted for OP	0.031 (0.012,0.049)	1.15E-03
Adjusted for CAD	0.025 (0.004,0.047)	2.05E-02

Abbreviations: FI, frailty index; FFS, Fried's frailty score; T2DM, type 2 diabetes mellitus; COPD, chronic obstructive pulmonary disease; OA, osteoarthritis; BMI, body mass index; OP, osteoporosis; CAD,coronary heart disease.

**Table 2:** Gene expression and frailty

Gene	Gene ID	beta (95%CI)	P_value
ANKRD55	ENSG00000164512	0 (0, 0.01)	4.36E-01
HLA-DPB2	ENSG00000224557	-0.02 (-0.03, -0.01)	2.65E-03
MLXIP	ENSG00000175727	-0.02 (-0.04, 0)	3.05E-02
